# Supplementary material for: Modeling glioblastoma heterogeneity as a dynamic network of cell states
Source: Mol Syst Biol. 2021 Sep 16;17(9):e10105. doi: 10.15252/msb.202010105 (PMC8444284; doi:10.15252/msb.202010105)
Supplement: Supplementary file 6 — Source Data for Figure 5 [file MSB-17-e10105-s004.zip › Figure5A_sourcedata/GSEA_3071/hallmarks_stateII.GseaPreranked.1623742536692/HALLMARK_COAGULATION.html]

Details for gene set HALLMARK\_COAGULATION[GSEA]

|  || Dataset | state23071 |
| Phenotype | NoPhenotypeAvailable |
| Upregulated in class | na\_neg |
| GeneSet | HALLMARK\_COAGULATION |
| Enrichment Score (ES) | -0.22974738 |
| Normalized Enrichment Score (NES) | -0.9738116 |
| Nominal p-value | 0.5065913 |
| FDR q-value | 0.6964319 |
| FWER p-Value | 1.0 |
Table: GSEA Results Summary

  

Fig 1: Enrichment plot: HALLMARK\_COAGULATION      
 Profile of the Running ES Score & Positions of GeneSet Members on the Rank Ordered List

  

| PROBE | GENE SYMBOL | GENE\_TITLE | RANK IN GENE LIST | RANK METRIC SCORE | RUNNING ES | CORE ENRICHMENT || 1 | F3 |  |  | 13 | 1.555 | 0.0722 | No |
| 2 | FBN1 |  |  | 143 | 0.972 | 0.0877 | No |
| 3 | SERPINE1 |  |  | 299 | 0.756 | 0.0862 | No |
| 4 | CSRP1 |  |  | 518 | 0.619 | 0.0626 | No |
| 5 | SH2B2 |  |  | 559 | 0.597 | 0.0817 | No |
| 6 | THBS1 |  |  | 624 | 0.568 | 0.0936 | No |
| 7 | DUSP14 |  |  | 643 | 0.555 | 0.1161 | No |
| 8 | PRSS23 |  |  | 666 | 0.546 | 0.1372 | No |
| 9 | LRP1 |  |  | 856 | 0.483 | 0.1141 | No |
| 10 | WDR1 |  |  | 908 | 0.469 | 0.1244 | No |
| 11 | MAFF |  |  | 986 | 0.447 | 0.1271 | No |
| 12 | ITGA2 |  |  | 1114 | 0.415 | 0.1160 | No |
| 13 | ITGB3 |  |  | 1325 | 0.374 | 0.0825 | No |
| 14 | CAPN2 |  |  | 1426 | 0.352 | 0.0750 | No |
| 15 | MMP14 |  |  | 2295 | -0.258 | -0.1260 | No |
| 16 | C1R |  |  | 2499 | -0.285 | -0.1621 | No |
| 17 | PROS1 |  |  | 2593 | -0.300 | -0.1704 | No |
| 18 | LAMP2 |  |  | 2724 | -0.321 | -0.1868 | No |
| 19 | CTSO |  |  | 2744 | -0.325 | -0.1757 | No |
| 20 | MMP2 |  |  | 2762 | -0.328 | -0.1640 | No |
| 21 | S100A13 |  |  | 2858 | -0.346 | -0.1706 | No |
| 22 | CAPN5 |  |  | 3018 | -0.378 | -0.1914 | No |
| 23 | ISCU |  |  | 3101 | -0.399 | -0.1922 | No |
| 24 | P2RY1 |  |  | 3189 | -0.426 | -0.1929 | No |
| 25 | FYN |  |  | 3262 | -0.444 | -0.1891 | No |
| 26 | MSRB2 |  |  | 3287 | -0.453 | -0.1730 | No |
| 27 | GSN |  |  | 3487 | -0.537 | -0.1960 | Yes |
| 28 | CD9 |  |  | 3567 | -0.581 | -0.1872 | Yes |
| 29 | HTRA1 |  |  | 3741 | -0.719 | -0.1948 | Yes |
| 30 | PLAT |  |  | 3766 | -0.752 | -0.1643 | Yes |
| 31 | DUSP6 |  |  | 3899 | -0.946 | -0.1508 | Yes |
| 32 | PDGFB |  |  | 3959 | -1.084 | -0.1127 | Yes |
| 33 | A2M |  |  | 3967 | -1.110 | -0.0606 | Yes |
| 34 | CLU |  |  | 4069 | -1.908 | 0.0071 | Yes |
Table: GSEA details [plain text format]

  

Fig 2: HALLMARK\_COAGULATION: Random ES distribution      
 Gene set null distribution of ES for **HALLMARK\_COAGULATION**
